# Differential Control of *i*NKT Cell Effector Lineage Differentiation by the Forkhead Box Protein O1 (Foxo1) Transcription Factor

**DOI:** 10.3389/fimmu.2019.02710

**Published:** 2019-11-21

**Authors:** Huishan Tao, Lei Li, Ying Gao, Zehua Wang, Xiao-Ping Zhong

**Affiliations:** ^1^Department of Pediatrics-Allergy and Immunology, Duke University Medical Center, Durham, NC, United States; ^2^Department of Gynecology and Obstetrics, Union Hospital, Tongji Medical College, Huazhong University of Science and Technology, Wuhan, China; ^3^Department of Breast and Thyroid Surgery, Union Hospital, Tongji Medical College, Huazhong University of Science and Technology, Wuhan, China; ^4^Department of Immunology, Duke University Medical Center, Durham, NC, United States; ^5^The Hematologic Malignancies and Cellular Therapy Research Program, Duke Cancer Institute, Duke University Medical Center, Durham, NC, United States

**Keywords:** invariant NK T cells, *i*NKT cells, Foxo1, T-bet, RORγt, T cell development, IFNγ, IL-17A

## Abstract

The invariant NKT (*i*NKT) cells recognize glycolipid antigens presented by the non-classical MHC like molecule CD1d. They represent an innate T-cell lineage with the ability to rapidly produce a variety of cytokines in response to agonist stimulation to bridge innate and adaptive immunity. In thymus, most *i*NKT cells complete their maturation and differentiate to multiple effector lineages such as *i*NKT-1, *i*NKT-2, and *i*NKT-17 cells that possess the capability to produce IFNγ, IL-4, and IL-17A, respectively, and play distinct roles in immune responses and diseases. Mechanisms that control *i*NKT lineage fate decisions are still not well understood. Evidence has revealed critical roles of Foxo1 of the forkhead box O1 subfamily of transcription factors in the immune system. However, its role in *i*NKT cells has been unknown. In this report, we demonstrate that deletion of Foxo1 causes severe decreases of *i*NKT cell total numbers due to impairment of late but not early *i*NKT cell development. Deficiency of Foxo1 results in decreases of *i*NKT-1 but increases of *i*NKT-17 cells. Our data reveal that Foxo1 controls *i*NKT effector lineage fate decision by promoting *i*NKT-1 but suppressing *i*NKT-17 lineages.

## Introduction

*i*NKT cells express TCRs that contain an invariant TCRVα14-Jα13 (iVα14) chain paired with limited numbers of TCRβ chain that recognize glycolipid antigens presented by the non-classic MHC-I like molecule CD1d. They play important roles in immune responses and tissue repair but also contribute to the pathogenesis of diseases (1–4). The iVα14 TCR signaling via multiple pathways such as RasGRP1-Ras-Erk1/2 pathway, the PKCθ-NFκB pathway, and mTOR is crucial for *i*NKT cell development in the thymus (2, 5–12). *i*NKT cells are traditionally defined into four developmental stages in the thymus: stage 0 (CD24^+^CD44^−^NK1.1^−^), stage 1 (CD24^−^CD44^−^NK1.1^−^), stage 2 (CD24^−^CD44^+^NK1.1^−^), and stage 3 (CD24^−^CD44^+^NK1.1^+^) (13, 14). Recently, *i*NKT cells have also been defined into multiple effector lineages based on expression of specific transcription factors and cytokines. These effector lineages identified thus far include IFN-γ-producing *i*NKT1, IL-4-producing *i*NKT2, IL-17A-producing *i*NKT17, *i*NKT10, Tfh-like *i*NKTfh, and Treg-like *i*NKT cells. *i*NKT1, *i*NKT2, *i*NKT17, and *i*NKTfh cells express T-bet, Gata3, RORγt, and Bcl6 that correspond to master transcription factors in individual T helper lineages, respectively (15–23). Stage 3 *i*NKT cells are virtually all *i*NKT1 cells (15, 24, 25), while *i*NKT17 cells reside in stage 2 CD44^+^NK1.1^−^ neuropilin-1^+^ ICOS^+^ cells (8, 18, 26–28). *i*NKT2 cells can be found in both stage 1 and stage 2 *i*NKT cells (28). Individual *i*NKT cell lineages exert distinct functions in immune responses and diseases. *i*NKT cell derived IL-4 participates in regulation of early germinal center B cell responses (29), liver regeneration and repair after injury (30), and adipocyte homeostasis (31). *i*NKT cell derived IL17 exacerbates airway hypersensitivity by recruiting neutrophils and inducing smooth muscle contraction (8, 20). *i*NKTfh cells secrets IL-21 and provides direct cognate help to antigen-specific B cells (16). Different from conventional αβT cells, most *i*NKT cells complete their effector lineage differentiation in the thymus and their relative abundance is influenced by genetic backgrounds, environmental factors, and intrinsic signal, metabolic, and transcriptional pathways. The mechanisms that control *i*NKT cell effector lineage fate decision are still not fully understood.

Foxo1 belongs to the Forkhead box “O” subfamily of transcription factors binding to a consensus DNA sequence 5′-(A/C)AA(C/T)A to regulate transcription of many genes involved in cell survival, cell cycle, differentiation, and cancer (32). Many mechanisms have evolved to regulate Foxo1 at both transcriptional and post transcriptional levels. A key mechanism is its phosphorylation by Akt and serum/glucocorticoid regulated kinase 1 (SGK1) on different sites that leads to its export from the nucleus and sequestration in the cytosol due to increased association to 14-3-3 proteins (33). Many studies have revealed important roles of the Foxo subfamily in the immune system (32, 34). Foxo1 promotes production of inflammatory cytokines in and regulates migration of dendritic cells (35, 36), is essential for B cell development and Ig class-switch (37–39), and regulates T cell differentiation and function (34). It controls T cell survival and homing via increasing IL7 receptor expression, CCR7, and L-selectin expression (40), plays important roles for regulatory T cell development and function (41–43), negatively controls Th17 cell differentiation (44–47) and Tfh differentiation (48–50), promotes Th9 responses (51–53), and regulates CD8 T cell effetor/memory differentiation (54–56). We report here that deficiency of Foxo1 does not grossly affect early *i*NKT cell development but severely impacts late *i*NKT cell development, manifested by severe reduction of *i*NKT1 cells but increases of *i*NKT17 cells. Our data suggest that Foxo1 is a critical regulator that controls *i*NKT1 and *i*NKT17 effector fate decision.

## Materials and Methods

### Mice

*Foxo1*^*f*/*f*^ and *Cd2iCre* mice were purchased from the Jackson Laboratory (57, 58). *Foxo1*^*f*/*f*^ had further backcrossed to C57BL6/J background. Two to ten-week-old *Foxo1*^*f*/*f*^-*Cd2iCre* and *Foxo1*^*f*/*f*^ or *Cd2iCre* littermate control mice were used for the experiments. For each experiment, one pair of test and control mice of the same sex littermates examined. All mouse experiments were performed according to protocols approved by the Duke University Institute Animal Care and Use Committee.

### Assessment of Foxo1 Recombination

Ten millions of viable CD4^+^CD8^+^ thymocytes from age- and sex-matched *Foxo1*^*f*/*f*^ and *Foxo1*^*f*/*f*^ *-Cd2iCre* mice were sorted on MoFlo Cell Sorter (Beckman Coulter) with post-sort purity of 98%. Genomic DNAs were extracted with phenol/chloroform, precipitated with 70% ethanol, dissolved in TE buffer (10 mM Tris-0.5 mM EDTA [pH 8]), and utilized as templates for PCR reaction. The forward and reverse primers for *Foxo1* were 5′-ACCACTCTGGACGGCATACT-3′ and 5′-TAACATAAAGGGAGATGAAGCA-3′, respectively. These two primers flank one of the two Loxp sites in the Foxo1 locus so that they did not amply a PCR product after Cre mediated recombination. Primers for Dgkz as control were 5′-AGAAAGCTGATCCCCCACAT-3′ (forward) and 5′-AGAGAGCGTCCTTCAAGAGG-3′ (reverse). PCR products were visualized after electrophoresis in 1% agarose gel.

### Flow Cytometry and Antibodies

Thymocytes, splenocytes, and liver mononuclear cells were prepared according to published protocols (9, 10). Cells were stained for surface markers with appropriate fluorochrome-conjugated antibodies in PBS containing 2% FBS on ice for 30 min followed by intracellular staining of transcription factors using the BD Bioscience Transcription Factor Buffer Set or Ki67 using the BD Bioscience Cytofix/Cytoperm™ solution according to the manufacturer's protocols. Data were collected using a BD LSRFortessa™ cytometer (BD Biosciences). PE- or allophycocyanin-labeled PBS57-loaded CD1d tetramers (CD1dTet) were provided by the NIH Tetramer Core Facility. Fluorochrome-conjugated anti-CD45.2 (clone 104), CD45.1 (A20), TCR-β (clone H57-597), NK1.1 (clone PK136), CD44 (clone IM7), CD24 (clone M1/69), CD11b (clone M170), CD11c (clone N418), F4/80 (clone BM8), B220 (clone RA3-6B2), TER119/Erythroid Cells (clone TER-119), CD4 (clone GK1.5), CD8a (clone 53-6.7), ICOS (clone C398.4A), T-bet (clone 4B10), IL7Rα (clone SB/199) were purchased from Biolegend; anti-GATA3 (clone L50-823), CD122 (clone TM-b1), RORγt (clone Q31-378), Streptavidin (BV711), and Ki67 were purchased from BD Biosciences; anti-PLZF (clone Mags.21F7) was purchased from eBioscience. Cell death was identified using the Live/Dead™ Fixable Violet Dead Cell Stain (Thermo Fisher Scientific). Reactive oxygen species (ROS) were detected with 2′,7′-dichlorodihydrofluorescein diacetate (H2DCFDA) (ThermoFisher). Goat anti-mouse IgG (H+L) antibody (Alexa Fluor 568) for detection of the anti-Ki67 antibody was purchased from Thermo Fisher Scientific. Data were analyzed using the FlowJo Version 9.2 software (Tree Star).

### Generation of Chimeric Mice

CD45.1^+^CD45.2^+^ WT mice in C57BL/6 background were irradiated with a single dose of 800 rad X-Ray and intravenously injected with 10–15 million of a mixture of BM cells from CD45.1^+^ WT mice and CD45.2^+^ *Foxo1*^*f*/*f*^ *-Cd2iCre* mice at 1:1 ratio. Recipient mice were euthanized and analyzed 8 weeks later.

### Statistical Analysis

Data were presented as mean ± SEM and analyzed for statistical differences using the Prism 5/GraphPad software. Comparisons were made using the two-tailed paired or unpaired Student *t*-test. Each pair represents age- and sex-matched littermates that were examined in a single experiment and is indicated by a connecting line between test and control mice. *P*-values less than 0.05 were considered significant (^*^p < 0.05, ^**^p < 0.01, ^***^p < 0.001).

## Results

### Impairment of *i*NKT Cell Development in *Foxo1^*f*/*f*^-Cd2iCre* Mice

**Figure 1:**
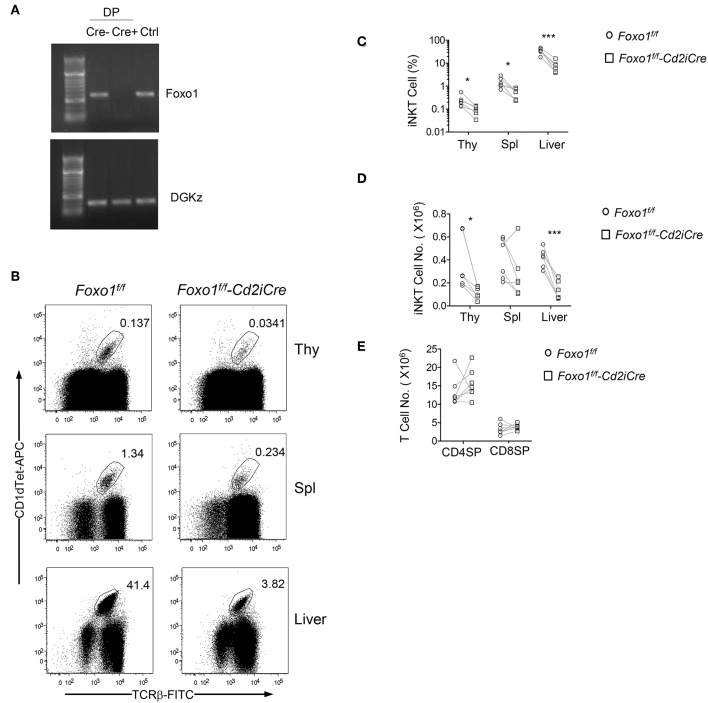
Severe decreases of *i*NKT cells in *Foxo1*^*f*/*f*^ *-Cd2iCre* mice. Six to ten weeks old *Foxo1*^*f*/*f*^ *-Cd2iCre* (Foxo1KO) *and Foxo1*^*f*/*f*^ or *Cd2iCre* controls (Ctrl) mice were analyzed for *i*NKT cells by flow cytometry. **(A)** Assessment of Cre mediated deletion in *Foxo1* gene in DP thymocytes. Cre mediated recombination causes deletion of the PCR template. Cre–: *Foxo1*^*f*/*f*^; Cre+: *Foxo1*^*f*/*f*^ *-Cd2iCre*; Ctrl: tail DNA from *Foxo1*^*f*/*f*^ *-Cd2iCre* mice. **(B)** TCRβ and CD1dTet staining of thymocytes, splenocytes, and liver mononuclear cells (MNCs). Live gated Lin-singlets are shown. **(C)** Percentages of *i*NKT cells in the indicated organ. **(D)** Numbers of *i*NKT cells in the indicated organs. **(E)** TCRβ^+^ SP thymocyte numbers. FACS plots are representative of five experiments. Scatter plots are pooled from five experiments **(C,D)** or seven experiments **(E)**. Each circle and square represents one WT and one Foxo1KO mice, respectively. Connecting lines indicate individual pairs of sex- and age-matched test and control mice in each experiment. One pair of mice was examined in each experiment. *p < 0.05; ***p < 0.001 determined by two-tail pair-wised Student *t*-test.

To investigate the role of Foxo1 in *i*NKT cell development, we bred *Foxo1*^*f*/*f*^ mice (57) with hCD2-iCre (*Cd2iCre)* mice (58) to generate *Foxo1*^*f*/*f*^ *-Cd2iCre* (Foxo1KO) mice. CD2iCre induces gene ablation of floxed genes in both T cells and B cells (58) and in CD4^+^CD8^+^ double positive (DP) thymocytes (Figure 1A). We used TCRβ and PBS-57 loaded CD1d tetramer (CD1dTet) to detect *i*NKT cells. In *Foxo1*^*f*/*f*^ *-Cd2iCre* mice, TCRβ^+^CD1dTet^+^ *i*NKT cell percentages and numbers were decreased 64.41% and 68.56%, respectively in the thymus compared with *Foxo1*^*f*/*f*^ controls (Figures 1B–D). Moreover, *i*NKT cell percentages and numbers were also reduced in the spleen and liver in *Foxo1*^*f*/*f*^ *-Cd2iCre* mice with the exception of splenic *i*NKT cells in one *Foxo1*^*f*/*f*^ *-Cd2iCre* mice due severe splenomegaly likely caused by defective function of regulatory T cells. In contrast, CD4^+^CD8^−^ single positive (SP) TCRβ^+^ and CD4^−^CD8^+^ SP TCRβ^+^ thymocyte numbers were similar between control and Foxo1KO mice (Figure 1E). Thus, Foxo1 deficiency resulted in severe impairment of *i*NKT cell but not conventional αβT cell development.

### Intrinsic Control of *i*NKT Development by Foxo1

**Figure 2:**
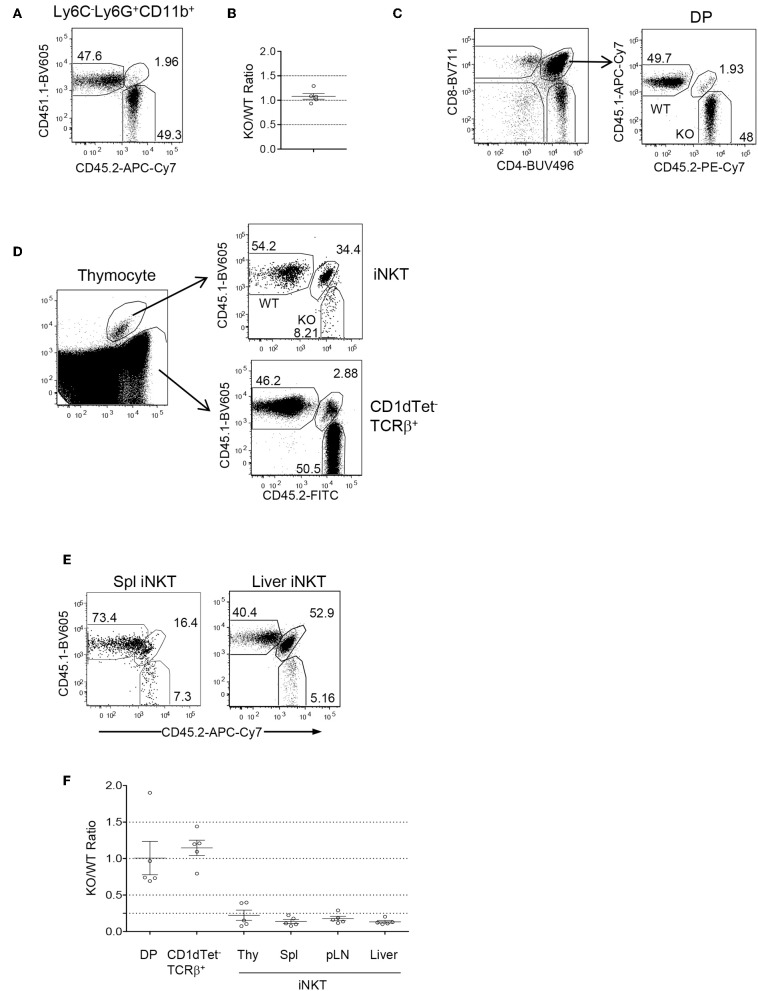
Intrinsic control of *i*NKT cell development by Foxo1. CD45.1^+^CD45.2^+^ WT mice were irradiated with 800 rad X-ray and intravenously injected with a mixture of CD45.1^+^ WT and CD45.2^+^ *Foxo1*^*f*/*f*^ *-Cd2iCre* BM cells at a 1:1 ratio. Recipient mice were euthanized and analyzed 2 months after reconstitution. **(A)** Representative FACS plots showing CD45.1 and CD45.2 staining in splenic Ly6C^−^Ly6G^+^CD11b^+^ neutrophils. **(B)** Scatter plots showing ratios of CD45.2^+^ to CD45.1^+^ neutrophils. **(C)** Representative FACS plots showing CD4 and CD8 staining of live gated thymocytes (left panel) and CD45.1 and 45.2 staining of CD4^+^CD8^+^ DP thymocytes (right panel). **(D)** Representative FACS plot showing CD1dTet and TCRβ staining (left panel) of live gated Lin-thymocytes and CD45.1 and 45.2 staining of gated CD1dTet^+^TCRβ^+^ *i*NKT cells (top right panel) and CD1dTet^−^TCRβ^+^ non-*i*NKT cells (bottom right panel). **(E)** Representative FACS plot showing CD45.1 and 45.2 staining of live gated CD1dTet^+^TCRβ^+^ *i*NKT cells from the spleen and liver. **(F)** CD45.2/CD45.1 ratios of indicated populations of cells. Data shown are representative or pooled from five experiments. One chimeric mouse was examined in each experiment.

Because Foxo1 was ablated in both T and B cell lineages and Foxo1 deficiency causes severe B cell developmental defects, impairment of regulatory T cell function, and Th lineage and CD8 effector/memory differentiation (32, 59), the severe decreases of *i*NKT cells could result from change of environment in these mice. To determine if abnormal *i*NKT cell development in *Foxo1*^*f*/*f*^ *-Cd2iCre* mice was autonomous, we generated mixed bone marrow (BM) irradiation chimeric mice by injecting a mixture of CD45.1^+^ WT and CD45.2^+^ *Foxo1*^*f*/*f*^ *-Cd2iCre* BM cells at a 1:1 ratio into sublethally irradiated CD45.1^+^CD45.2^+^ recipient mice. Two months after reconstitution, recipient mice contained about 1:1 ratio of CD45.2^+^ and CD45.1^+^ CD11b^+^Ly6G^+^Ly6C^−^ neutrophils in the spleen (Figures 2A,B) suggesting equal reconstitution of these two types of hematopoietic stem cells (HSCs). Additionally, the ratios of CD45.2^+^ to CD45.1^+^ CD4^+^CD8^+^ double positive (DP) thymocytes, the immediate precursors of *i*NKT cells, and non-*i*NKT TCRβ^+^ conventional T cells were also 1:1 (Figures 2C,D,F). However, the CD45.2^+^ Foxo1KO to CD45.1 WT ratios for *i*NKT cells were drastically decreased to 0.2:1 (Figures 2D,F). In contrast, the ratio for CD1dTet^−^TCRβ^+^ conventional αβT cells in the thymus were not decreased (Figures 2D,F). Consistent with severe defects in *i*NKT cell generation, very few *i*NKT cells in the spleen and liver in the recipients were derived from Foxo1KO HSCs (Figures 2E,F). Thus, Foxo1 deficiency intrinsically inhibited *i*NKT but not conventional αβT cell development.

### Foxo1 Deficiency Caused Impairment of Late Stage *i*NKT Cell Development

**Figure 3:**
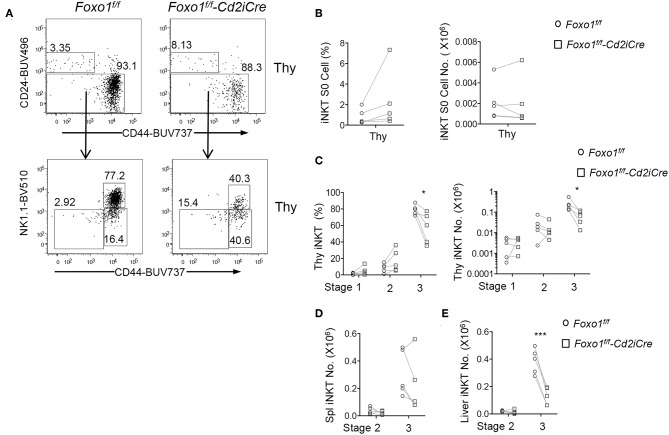
Impaired late stage *i*NKT cell development in *Foxo1*^*f*/*f*^ *-Cd2iCre* mice. Six to ten weeks old *Foxo1*^*f*/*f*^ *-Cd2iCre and* control mice were analyzed similar to Figure 1 with additional antibodies for CD24, CD44, NK1.1, and CD11b, CD11, F4/80, Ter119 for lineage gating. **(A)** Top panels: CD24 vs. CD44 staining of live gated Lin^−^ *i*NKT cells in the thymus. Bottom panels, CD44 vs. NK1.1 staining on CD24^−^ *i*NKT cells. **(B)** Stage 0 *i*NKT percentages and numbers in the thymus. **(C)** Stages 1, 2, and 3 *i*NKT cell percentages and numbers in the thymus. **(D)** Stages 2 and 3 *i*NKT cell numbers in the spleen. **(E)** Stages 2 and 3 *i*NKT cell numbers in the liver. Data shown are representative or pooled from five experiments. Connecting lines indicate individual pairs of sex- and age-matched test and control mice in each experiment. *p < 0.05; ***p < 0.001 determined by two-tail pair-wised Student *t*-test.

*i*NKT cell development in the thymus is traditionally defined sequentially into CD24^+^CD44^−^NK1.1^−^ stage 0, CD24^−^CD44^−^NK1.1^−^ stage 1, CD24^−^CD44^+^NK1.1^−^ stage 2, and CD24^−^CD44^+^NK1.1^+^ stage 3 (13, 14). In *Foxo1*^*f*/*f*^ *-Cd2iCre* mice, stage 0 *i*NKT cell percentages were increased but numbers were not altered compared with control mice (Figures 3A,B). Stages 1 and 2 *i*NKT cell percentages displayed increased trend, although not statistically significant. However, their numbers were not obviously altered (Figures 3A,C). Importantly, stage 3 *i*NKT cell percentages and numbers were both decreased in *Foxo1*^*f*/*f*^ *-Cd2iCre* thymus (Figures 3A,C). In Foxo1KO spleen, stage 2 *i*NKT cell numbers were similar to WT control but stage 3 showed decreased except one mouse (Figure 3D), which contained increased stage 3 *i*NKT cells due to considerable enlargement of the spleen that was likely caused defects in regulatory T cells and lymphoproliferative disorders. In Foxo1KO liver, stage 2 *i*NKT cell numbers were also similar to WT control but stage 3 *i*NKT cells were obviously decreased (Figure 3E). Thus, Foxo1 deficiency appears to selectively affect late stage *i*NKT cell development.

### Intrinsic Promotion of Late Stage *i*NKT Maturation by Foxo1

**Figure 4:**
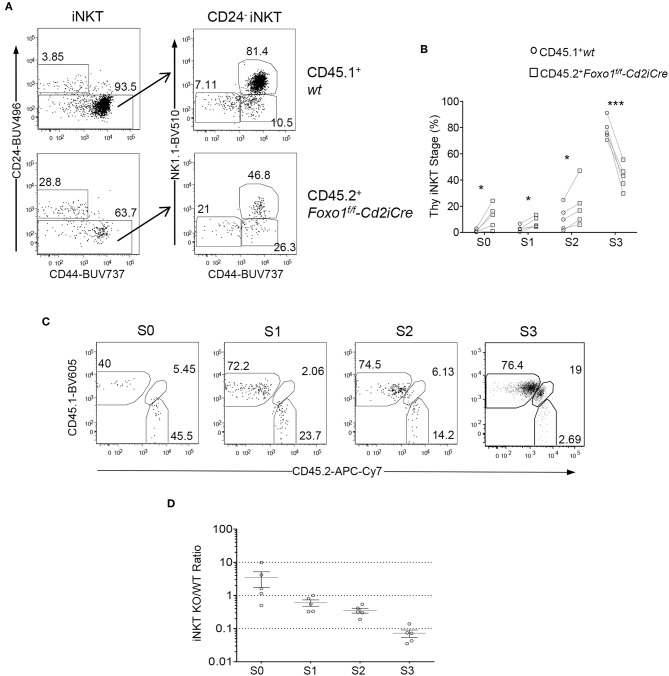
Intrinsic control of late stage *i*NKT cell development by Foxo1. Thymocytes and splenocytes and liver MNCs from mixed BM chimeric mice as described in Figure 2 were similarly analyzed. **(A)** Representative FACS plots showing CD24 and CD44 staining in live gated Lin-CD45.1^+^ WT and CD45.2^+^ Foxo1KO thymic *i*NKT cells (left panels) and CD44 and NK1.1 staining in the CD24^−^ population of *i*NKT cells. **(B)** Scatter plots showing percentages of *i*NKT cell developmental stages. **(C)** Representative FACS plots showing CD45.1 and CD45.2 staining on stage 0–3 *i*NKT cells. **(D)** Scatter plots showing CD45.2/CD45.1 ratios of individual *i*NKT cell developmental stages. Connecting lines indicate CD45.1 WT and CD45.2 Foxo1KO *i*NKT cells in individual mice. One chimeric mouse was examined in each experiment. *p < 0.05; ***p < 0.001 determined by two-tail pair-wised Student *t*-test.

We further examined whether Foxo1 intrinsically promotes late stage *i*NKT cell maturation using the mixed BM chimeric mice described in Figure 2. Within CD45.2^+^ Foxo1KO *i*NKT cells, the percentages of stages 0, 1, and 2 were increased 11.08, 2.54, and 1.87 folds compared with CD45.1^+^ WT *i*NKT cells, respectively; but stage 3 were drastically decreased (Figures 4A,B), indicating an intrinsic role of Foxo1 for *i*NKT cell terminal maturation to stage 3. Because *i*NKT cells were predominantly stage 3 and its dramatic decreases could affect the percentages of stage 0–2 *i*NKT cells, we further calculated CD45.2 Foxo1KO/CD45.1 WT ratios of each stage of *i*NKT cells in individual recipient mice. As shown in Figures 4C,D, the ratios of CD45.2/CD45.1 of stage 0 *i*NKT cell were increased to 3.4:1 compared with the ratios of DP thymocytes. But the ratios of stages 1, 2, and 3 were progressively decreased from 0.60:1, to 0.35:1, and finally to 0.073:1. These results suggested that Foxo1 deficiency exerted weak inhibition on stage 1 and stage 2 *i*NKT cell maturation but much strong impact on stage 3 *i*NKT cell maturation. The increases of stage 0 ratios could be caused by enhanced early *i*NKT cell generation and/or blockade of stage 0 to stage 1 *i*NKT cell transition in the absence of Foxo1. Thus, Foxo1 intrinsically promoted *i*NKT cell maturation, particularly late stage maturation.

### Differential Effects of Foxo1 Deficiency on *i*NKT Effector Lineage Differentiation

**Figure 5:**
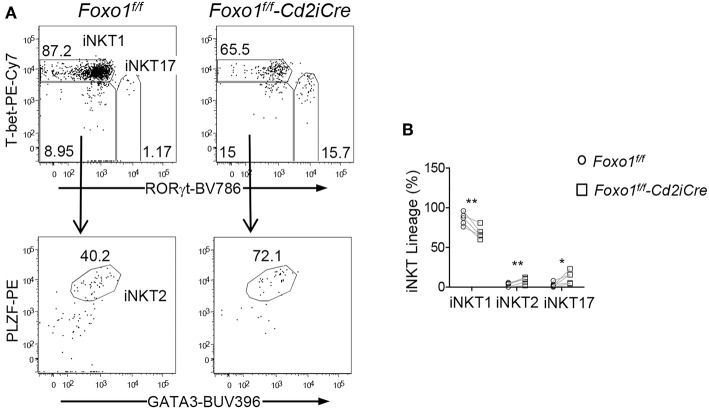
Selective impairment of *i*NKT1 differentiation in Foxo1 deficient mice. **(A)** Top panels: T-bet and RORγt staining of thymic *i*NKT cells. Bottom panels: PLZF and GATA3 staining of RORγt^−^T-bet^−^ *i*NKT cells. **(B)** Percentages of *i*NKT effector lineages. Data shown are representative or pooled from five experiments. Connecting lines indicate individual pairs of sex- and age-matched test and control mice in each experiment. One pair of mice was examined in each experiment. *p < 0.05; **p < 0.01 determined by two-tail pair-wised Student *t*-test.

*i*NKT cells differentiate to multiple effector lineages in the thymus that can be defined based expression transcriptional factors/repressors PLZF, GATA3, T-bet, and RORγt (15). Within *i*NKT cells from *Foxo1*^*f*/*f*^ *-Cd2iCre* thymus, the percentages of PLZF^+^GATA3^+^ *i*NKT2 and RORγt^+^T-bet^−^ *i*NKT17 cells were increased but RORγt^−^T-bet^+^ *i*NKT1 cells were decreased (Figures 5A,B). Thus, Foxo1 deficiency greatly inhibited *i*NKT1 development, which was consistent with severe decreases of stage 3 *i*NKT cells that are mostly *i*NKT1 cells.

### Intrinsic Control of *i*NKT Effector Lineage Differentiation by Foxo1

**Figure 6:**
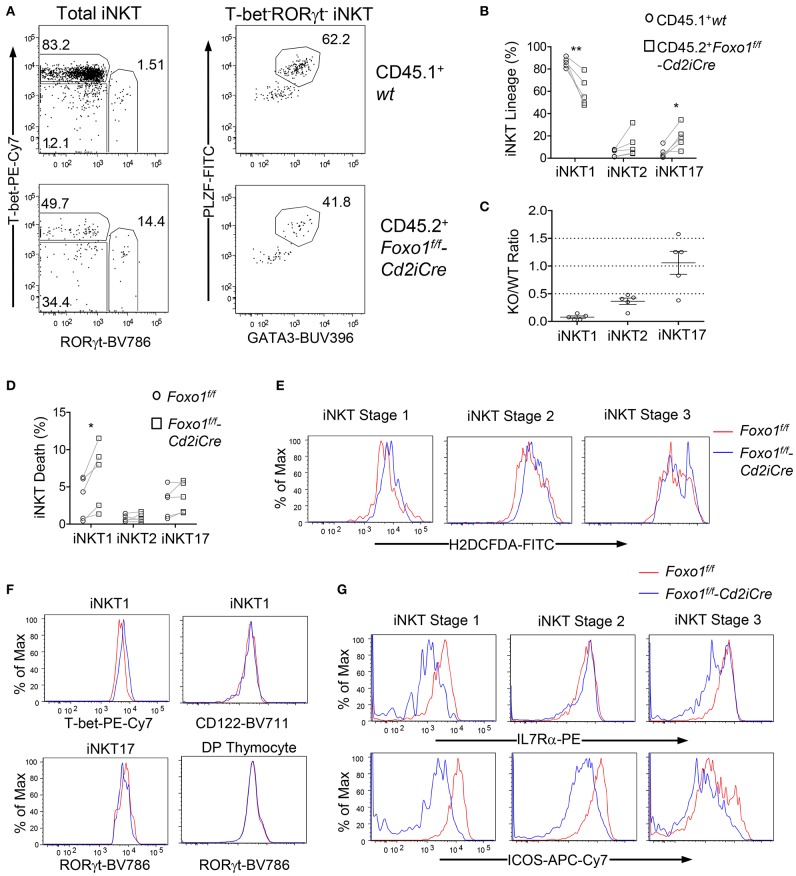
Intrinsic control of *i*NKT cell effector lineage differentiation by Foxo1. **(A–C)** Thymocytes from mixed BM chimeric mice as described in Figure 2 were similarly analyzed. **(A)** Representative FACS plots showing RORγt and T-bet staining in gated CD45.1^+^ WT and CD45.2^+^ Foxo1KO *i*NKT cells (left panels) and PLZF and GATA3 staining in gated RORγt^−^T-bet^−^ *i*NKT cells. **(B)** Scatter plots showing percentages of *i*NKT effector lineages. **(C)** Scatter plots showing CD45.2 Foxo1KO/CD45.1WT ratios of individual *i*NKT effector lineages. Data shown are representative or pooled from five experiments. Connecting lines indicate CD45.1 WT and CD45.2 Foxo1KO *i*NKT cells in individual mice. One chimeric mouse was examined in each experiment.*p < 0.05; **p < 0.01 determined by two-tail pair-wised Student *t*-test. **(D–G)** Two to three weeks old *Foxo1*^*f*/*f*^ *-Cd2iCre and Foxo1*^*f*/*f*^ mice were analyzed for thymic *i*NKT cells by flow cytometry. **(D)** Death rates of *i*NKT effector lineages detected by Live/Dead™ Fixable Violet Dead Cell Stain. *N* = 5. *p < 0.05 determined by two-tail pair-wised Student *t*-test. **(E)** Overlaid histograms show ROS levels in stages 1–3 *i*NKT cells. **(F)** Overlaid histograms show T-bet and CD122 levels in *i*NKT1 cells and RORγt levels in *i*NKT17 cells and in DP thymocytes. **(G)** Overlaid histograms show IL7Rα and ICOS levels in stages 1–3 *i*NKT cells. Data in **(E–G)** are representative of three experiments.

Because *i*NKT effector lineages may compete with each other during development and are influenced by thymic environment, we further examined whether Foxo1 intrinsically controls *i*NKT cell effector lineage differentiation using the mixed BM chimeric mice described in Figure 2. Similar to data shown in Figure 5, the percentages of *i*NKT1 were decreased but *i*NKT17 and, to certain extent, *i*NKT2 were increased within CD45.2^+^ Foxo1KO *i*NKT cells (Figures 6A,B). Additionally, the Foxo1KO to WT ratios of *i*NKT1, *i*NKT2, and *i*NKT17 cells were 0.077:1, 0.36:1, and 1.1:1, respectively (Figure 6C). Because the Foxo1KO/WT *i*NKT17 ratios (1.1:1) were greater than stage 2 *i*NKT ratios (0.35:1, Figure 4D), it suggested that Foxo1KO *i*NKT17 differentiation was enhanced and that Foxo1 deficiency intrinsically inhibited *i*NKT1 but promoted *i*NKT17 differentiation.

In Foxo1KO mice, *i*NKT1 cells but not *i*NKT2 or *i*NKT17 cells were prone to death compared with WT *i*NKT cells (Figure 6D). ROS levels were weakly increased in stage 3 *i*NKT cells (Figure 6E), which might contribute increased death and decreases of *i*NKT1 cells as most stage 3 *i*NKT cells are *i*NKT1 cells. Foxo1 inhibits T-bet expression in NK cells (60). Within Foxo1KO *i*NKT1 cells, T-bet expression was increased compared with WT controls, suggesting that Foxo1 also inhibited T-bet expression in *i*NKT cells (Figure 6F). Additionally, CD122, the IL2/15Rβ chain that mediates IL15 signal to positively regulate T-bet expression and to promote *i*NKT1 differentiation and survival (61, 62), was expressed at similar levels between WT and Foxo1KO *i*NKT1 cells (Figure 6F), suggesting that the reduction of Foxo1KO *i*NKT1 cells was unlikely due to altered T-bet or CD122 expression. RORγt is crucial *i*NKT17 differentiation (18). RORγt levels were not obviously changed in Foxo1KO *i*NKT17 cells or in DP thymocytes (Figure 6F). IL7 receptor (IL7R) signal and ICOS costimulatory signal promote *i*NKT17 lineage differentiation/homeostasis (8, 61, 63). However, both IL7Rα and ICOS levels were reduced in *i*NKT cells at different stages (Figure 6G), suggesting that Foxo1 deficiency alleviated the requirement of these signals for *i*NKT17 cells and might promote *i*NKT17 lineage via other mechanisms.

## Discussion

*i*NKT cells differentiate to multiple effector lineages that play distinct roles in immune responses and diseases. Evidence suggests that *i*NKT1 and *i*NKT17 are competing lineages during *i*NKT effector differentiation. Both intrinsic program and environmental factors are both involved in control the balance between *i*NKT1 and *i*NKT17 cells (8, 15, 63–67). In this report, we demonstrated that deficiency of Foxo1 intrinsically inhibited *i*NKT1 but enhanced *i*NKT17 differentiation without obviously affecting early *i*NKT cell development, suggesting that Foxo1 controls *i*NKT effector lineage fate decision.

In *Foxo1*^*f*/*f*^ *-Cd2iCre* mice, stage 0, 1, and 2 *i*NKT cell numbers are not obviously different from WT control mice and only stage 3 *i*NKT cell numbers and percentages were decreased. In mixed BM chimeric mice reconstituted with *Foxo1*^*f*/*f*^ *-Cd2iCre* and congenic WT BM cells, Foxo1KO to WT ratios of stage 0 *i*NKT cells are slightly increased compared with the ratios of CD4^+^CD8^+^ thymocytes. Thus, Foxo1 is dispensable for early *i*NKT cell development. However, other members of the Foxo subfamily are also expressed in developing thymocytes, our data do not rule out the possibility that those factors may function redundantly with Foxo1 and compensate for its deficiency during early *i*NKT cell development. Further studies with compound deficiency of Foxo1 and other family members are needed to fully rule out a role of Foxo1 in early *i*NKT cell development.

Foxo1 deficiency leads to considerable decreases of CD44^+^NK1.1^+^T-bet^+^ *i*NKT1 cells but increases of CD44^+^NK1.1^−^RORγt^+^ *i*NKT17 cells. *i*NKT1 cell percentages and numbers are both decreased, revealed a positive role of Foxo1 in promoting *i*NKT1 differentiation/ maintenance. The increases of *i*NKT17 cells in the absence of Foxo1 are not solely resulted from decreases of *i*NKT1 cells and consequent increases of *i*NKT17 ratios. The Foxo1KO to WT ratios of *i*NKT17 cells are significantly overrepresented compared with those of stage 1 and 2 *i*NKT cells in mixed BM chimeric mice, indicating that Foxo1 negatively controls *i*NKT17 differentiation. Thus, Foxo1 plays an intrinsic role in controlling *i*NKT1/17 effector fate decision and may rheostat the balance between these two *i*NKT effector lineages.

Numerous mechanisms have been found to control *i*NKT1/17 lineage differentiation. Many transcription factors and regulators such as cMAF, Runx1, MED2/3, and NKAP are important for *i*NKT17 differentiation (68–74), while the epigenetic modifiers of the TET-family dioxygenases inhibit *i*NKT17 but promote *i*NKT1 differentiation (67). Foxo1 deficient *i*NKT1 but not *i*NKT2/17 cells were prone to death and contained increased ROS, suggesting that Foxo1 might regulate ROS production to promote *i*NKT1 cell survival. IL15R signal is critical for *i*NKT1 cell differentiation and homeostasis in part by increasing T-bet expression (61, 62). We have found IL15Rβ chain expression is not reduced in Foxo1KO *i*NKT1 cells. Thus, Foxo1 deficiency may cause *i*NKT1 defect via mechanisms other than altered expression of T-bet or CD122. IL7R signal and ICOS costimulatory signal promote *i*NKT17 cell homeostasis (8, 61, 63). Both IL7Rα and ICOS levels were decreased in Foxo1KO *i*NKT cells. In conventional T cells, Foxo1 binds to *Il7ra* and *Icos* loci to promote their transcription (48, 75). Foxo1 may function similarly to promote expression of these molecules in *i*NKT cells. However, it is unlikely that the decreased expression of IL7R and ICOS causes enhanced *i*NKT17 differentiation in Foxo1KO mice. Although ICOS and IL7R are not crucial for *i*NKT1 differentiation, our data do not rule out the possibility that decreased expression of these molecules may impair *i*NKT1 lineage differentiation/homeostasis in the context of Foxo1 deficiency.

Foxo1 inhibits Th17 differentiation and IL-17A expression by directly suppressing transcription of RORγt and IL-23R expression and by interacting with RORγt (44, 47, 76). Although such mechanisms could operate similarly *i*NKT cells, we do not observe increased RORγt protein levels in Foxo1KO *i*NKT17 cells. It is interesting to note that Foxo1 inhibits T-bet mediated effector differentiation of CD8 T cells and Th1 differentiation (55, 77) and binds to the *Tbx21* promoter to inhibit T-bet expression in NK cells (60). In Foxo1 deficient *i*NKT1 cells, T-bet levels were increased, suggesting that Foxo1 may also directly suppress T-bet expression in *i*NKT cells. However, increased T-bet expression should not be the reason for deficiency of *i*NKT1 cells in Foxo1KO mice.

Foxo1 is regulated by multiple mechanisms. Akt, SGK1, and the serine/threonine kinase CK2 phosphorylate Foxo1 to inhibit its nuclear localization and activity. mTOR regulates *i*NKT1 and *i*NKT17 differentiation via mTOR complex 1 (mTORC1) and mTORC2 and their tight regulation by the tumor suppressor TSC1 ensures proper *i*NKT1/17 balance (8, 66, 78, 79). mTORC2 phosphorylates Akt and SGK1 to enhance their enzyme activities (80–82). Deficiency of either Akt2 or mTORC2 causes decreases of *i*NKT17 cells, which correlates with decreased Foxo1 phosphorylation or increased Foxo1 nuclear localization (66, 83). The data we report here provide direct evidence that Foxo1 plays critical roles in *i*NKT cell development and effector lineage differentiation. Although the importance of SGK1 and CK2 in *i*NKT cells has been unclear, both SGK1 and CK2 promote Th17 differentiation at least partially through inhibition of Foxo1 (45, 84, 85). Our data together with these observations indicate that Foxo1 may integrate signals from mTORC2/Akt/SGK1 and other enzymes to control *i*NKT cell effector lineage fate decision.

## Data Availability Statement

The datasets generated for this study are available on request to the corresponding author.

## Ethics Statement

The protocol was approved by the Duke University Institute Animal Care and Use Committee.

## Author Contributions

HT and LL designed and performed experiments, analyzed data, and were involved in manuscript preparation. YG and ZW were involved in data analyses and manuscript preparation. X-PZ conceived the project, designed experiments, analyzed data, and wrote the manuscript.

### Conflict of Interest

The authors declare that the research was conducted in the absence of any commercial or financial relationships that could be construed as a potential conflict of interest.
